# mGluR2/3 blockade produces rapid and long-lasting reversal of anhedonia caused by chronic stress exposure

**DOI:** 10.1186/2049-9256-1-15

**Published:** 2013-09-17

**Authors:** Jason M Dwyer, Ashley E Lepack, Ronald S Duman

**Affiliations:** Laboratory of Molecular Psychiatry, Center for Genes and Behavior, Departments of Psychiatry and Neurobiology, Yale University School of Medicine, 34 Park Street, Room S308, New Haven, CT 06508 USA

**Keywords:** mGluR2/3, Stress, Depression, Antidepressant, Ketamine, LY341495

## Abstract

**Background:**

Depression is a prevalent neuropsychiatric disorder that affects an estimated 350 million people worldwide. Currently available treatments for depression are lacking in both speed of onset and efficacy. Recent pharmacological efforts have targeted the glutamatergic neurotransmitter system using the N-methyl-D-aspartate (NMDA) receptor antagonist ketamine to produce rapid and robust antidepressant effects, however the widespread clinical use of ketamine is limited due to side effects and abuse liability. More recently, work evaluating metabotropic mGluR2/3 receptor antagonists has demonstrated many similarities with ketamine.

**Methods:**

Male, Sprague–Dawley rats were exposed to a chronic unpredictable stress paradigm, which produces decreased sucrose preference, a measure of anhedonia. Rats were then treated with vehicle or a single injection of the mGluR2/3 antagonist LY341495 (3 mg/kg, i.p.) and tested at 24 hrs, 48 hrs or 10 days after a single treatment.

**Results:**

We demonstrate that a single treatment with LY341495 produces a rapid (within 1–2 days) and long-lasting (10 days) reversal of anhedonia caused by chronic unpredictable stress in rats. This model provides a rigorous test of rapid-acting agents as typical antidepressants require several weeks of treatment to produce a response.

**Conclusions:**

These data suggest that LY341495 has the ability to produce rapid and robust antidepressant effects similar to ketamine. Together, the results highlight the potential for similar compounds to produce rapid and lasting efficacy for the treatment of depression.

## Background

Major Depressive Disorder (MDD) is a debilitating neuropsychiatric disorder that affects nearly one fifth of the US population [1], and according to the World Health Organization (WHO), affects an estimated 350 million people worldwide, making it the leading cause of disability. Currently available antidepressants target monoaminergic neurotransmitter systems, however these agents produce limited efficacy (~33% initial response rate) and require several weeks to months of chronic treatment. Development of novel agents that produce a rapid and robust antidepressant response represents a major unmet medical need for the treatment of MDD.

The discovery that the non-competitive N-methyl-D-aspartate (NMDA) receptor antagonist ketamine produces rapid antidepressant effects in humans (within 2 hours) that last roughly one week after a single intravenous administration has generated interest in targeting the glutamatergic system for the treatment of MDD [2, 3]. Much like studies in humans, preclinical studies have demonstrated that ketamine also produces rapid antidepressant effects in rodent models of depression, such as the chronic unpredictable stress (CUS)-anhedonia paradigm, which can detect agents with rapid onset of action [4]. In addition, evidence suggests that ketamine has the unique ability to rapidly reverse losses of excitatory spine synapses in the medial prefrontal cortex (mPFC) within 24 hrs following exposure to three weeks of CUS [4]. Furthermore, this reversal of the behavioral and neuronal deficits produced by CUS requires signaling through the mechanistic target of rapamycin complex 1 (mTORC1) pathway [4, 5], which is a ubiquitously expressed serine/threonine kinase pathway involved in regulation of cell growth and protein translation.

Given the side effect profile and abuse potential of ketamine, its clinical use is limited. Therefore, efforts have focused on developing drugs that target the glutamatergic system to produce ketamine-like rapid antidepressant responses without the side effects or abuse liability. Recently, research has focused on targeting subtypes of metabotropic glutamate receptors (mGluRs). One of the most widely studied is the mGluR Group II. Group II mGluRs consist of mGluR2 and mGluR3 subtypes and are seven transmembrane G-protein coupled receptors that negatively regulate adenylyl cyclase and function to decrease neurotransmitter release. The mGluR2 receptor is located pre- and post-synaptically [6] but is thought to be predominantly located at the pre-terminal portion of axons [7] where it functions as an autoreceptor. While mGluR2 expression seems to be confined to neurons [8], mGluR3 receptors, which are also pre- and post-synaptic, are located on neurons as well as glia [9]. Studies have demonstrated that these receptors are localized in regions associated with depression and emotional responses, such as the mPFC and hippocampus [10]. Additionally, a number of studies have demonstrated that antagonists of Group II mGluRs produce robust antidepressant responses in acute rodent models [11, 12].

Recently, work by a number of labs has demonstrated similarities between ketamine and mGluR2/3 antagonists. Much like ketamine, mGluR2/3 antagonists produce rapid and transient increases in glutamate release in the mPFC [13, 14]. Blockade of post-synaptic AMPA receptors blocks the antidepressant effects of both ketamine and mGluR2/3 antagonists in rodent models [15, 16]. Interestingly, much like ketamine, the behavioral antidepressant effects of mGluR2/3 antagonists require signaling through the mTORC1 pathway [17, 18]. The selective mGluR2/3 antagonist LY341495 increases activity of mTORC1 and its two major downstream substrates, p70 S6 kinase and 4E-BP1 [17]. Furthermore, this increase in mTORC1 pathway signaling is associated with increases in critical synaptic proteins PSD-95, GluR1 and synapsin I [17].

These data suggest that, much like ketamine, mGluR2/3 antagonism may have the ability to rapidly reverse the behavioral deficits produced by CUS. To examine this possibility we employed a CUS model that results in decreased preference for a sweetened solution (1% sucrose). Reduced sucrose preference is a measure of anhedonia, a core symptom of depression, which requires several weeks of chronic treatment with typical antidepressants to reverse.

## Methods

Adult male Sprague–Dawley rats (Charles River) weighing ~300 g at the beginning of the experiment were randomly assigned to control or CUS groups and exposed to CUS. In brief, rats were exposed to 2 stressors per day consisting of overnight isolation, manipulations of light cycle (i.e. light on overnight or 3 hr light off during the light cycle), stroboscopic light overnight, overnight food and water deprivation, overnight crowding, overnight cage tilt, overnight wet bedding, 1 hr restraint, 10 min swim in cold water (18°C), 1 hr cold exposure (4°C), or 1 hr shaking on a rotator. Control rats were handled daily. After 28 days of stress, rats were assessed for sucrose preference. Rats exposed to CUS showed the expected lack of preference for 1% sucrose compared to non-stressed control rats (78% preference for control vs. 48% preference for CUS *P* < 0.05, *t*-test). Control and CUS groups were then divided and counterbalanced for vehicle and drug treatment groups and given a single intraperitoneal (i.p.) injection of vehicle (saline) or 3 mg/kg of the selective mGluR2/3 antagonist LY341495 (Tocris BioScience). Rats were then tested 24 hrs, 48 hrs, and 10 days after drug administration. All experiments were conducted in accordance with the NIH guidelines for the care and use of laboratory animals and were approved by the Yale University Institutional Animal Care and Use Committee (IACUC).

## Results and discussion

**Figure 1:**
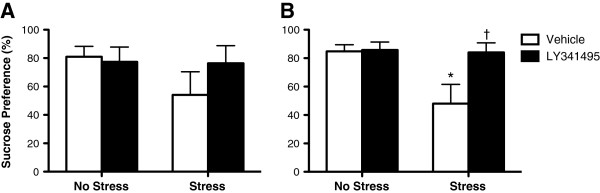
**LY341495 produces a rapid antidepressant effect. (A)** LY341495 produced a trend toward a reversal of stress-induced decreased sucrose preference at 24 hrs post-LY341495 treatment (*not significant*). **(B)** LY341495 reversed the stress-induced decrease in sucrose preference 48 hrs after a single treatment, indicating a rapid antidepressant effect. *P < 0.01 compared to No Stress/Vehicle; †*P* < 0.01 compared to Stress/Vehicle group; Stress x LY341495 interaction *P* = 0.05 (*two-way ANOVA; LSD post-hoc tests*).

Results demonstrate a trend toward reversal of the decreased sucrose preference resulting from CUS exposure at 24 hrs and a significant reversal at 48 hrs post LY341495 treatment (Figure 1). These data suggest that LY341495 produces a rapid reversal of CUS-induced anhedonia within days after a single treatment, an antidepressant effect that requires weeks of administration of typical antidepressants. This rapid effect of LY341495 is similar to that observed following ketamine treatment as previous studies have shown a reversal of the deficit in sucrose preference within 24 hrs of ketamine treatment [4]. While we failed to find statistical significance at the 24 hr test following LY341495 treatment, this is likely due to the high variability within groups. No significant effects of LY341495 on water consumed or total fluid consumed were observed.

**Figure 2:**
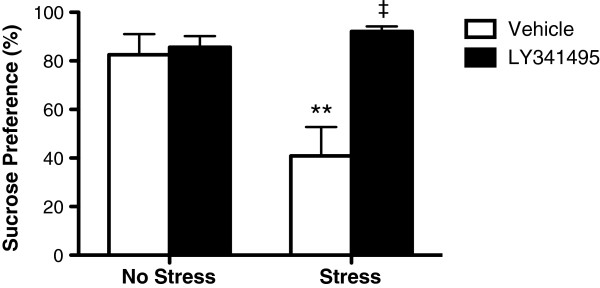
**The antidepressant effect of a single injection of LY341495 was sustained for 10 days.** **P < 0.001 compared to No Stress/Vehicle; ‡*P* < 0.001 compared to Stress/Vehicle group; Stress x LY341495 interaction *P* < 0.01 (*two-way ANOVA; LSD post-hoc tests*).

To assess whether LY341495 also produces long-lasting antidepressant effects, the same rats were tested 10 days after the initial injection of LY341495 with continued exposure to CUS. Much like the data observed at 48 hours post-injection, CUS-induced anhedonia was still significantly blocked in rats that were administered LY341495 10 days earlier, demonstrating a long-lasting antidepressant response following a single treatment (Figure 2).

The CUS model is an ideal rodent model for testing putative rapid-acting antidepressants. Given that typical antidepressants, such as imipramine, fluoxetine, and other monoamine reuptake inhibitors, require several weeks of chronic treatment [19], the CUS model allows for detection of rapid-acting agents. For example, typical antidepressants fail to reverse the effects of chronic stress following 1 week of treatment [19]. Taken together, the data presented here demonstrate that mGluR2/3 antagonists are capable of producing rapid and long-lasting antidepressant effects. Given that LY341495 produces a very similar biochemical signature to ketamine (i.e. increases in mTORC1 signaling and synaptic protein content), which is required for the antidepressant effects of mGluR2/3 blockade, drugs targeting mGluR2/3 receptors hold promise as future antidepressants. It is interesting to note that antidepressant responses to LY341495 are observed long after the half-life of the drug (~45 min following i.p. injection) [20], suggesting that it is likely that mGluR2/3 blockade produces morphological and functional changes in mPFC neurons reminiscent of those produced by ketamine. Future experiments measuring spine density and excitatory postsynaptic potentials will be necessary to determine if this is the case. Additionally, it will be interesting to see if LY341495 reverses HPA axis dysregulation observed following chronic stress. Given that the goal of drug discovery for MDD is to identify agents that lack ketamine’s side-effect and abuse potential, future studies will also need to address these issues with putative mGluR2/3 antidepressants. Future experiments with novel pharmacological tools will be necessary to identify the specific roles of mGluR2 and mGluR3 receptors in mediating these rapid responses.

## Conclusion

The mGluR2/3 antagonist LY341495 produces rapid and long-lasting antidepressant effects in the rodent CUS model. These data suggest that drugs targeting mGluR2/3 receptors may hold promise for rapid and lasting treatment of patients suffering from MDD.
